# Relationship between cognitive appraisals of symptoms and negative mood for subtypes of irritable bowel syndrome

**DOI:** 10.1186/1751-0759-2-9

**Published:** 2008-04-08

**Authors:** Nagisa Sugaya, Shinobu Nomura

**Affiliations:** 1 Faculty of Human Sciences, Waseda University, Saitama, Japan

## Abstract

**Background:**

The onset and course of irritable bowel syndrome (IBS) are strongly influenced by psychological factors, and treatment often includes cognitive-behavioral therapy. We conducted a study of the relationships between cognitive appraisal of IBS symptoms and negative mood for the subtypes of IBS.

**Method:**

The participants were 1087 college students who completed a set of questionnaires that included the Rome II Modular Questionnaire, Self-reported IBS Questionnaire, Cognitive Appraisal Rating Scale, and the Hospital Anxiety and Depression Scale.

**Results:**

The participants included 206 individuals with IBS; 61 had diarrhea-predominant IBS (IBSD) and 45 had constipation-predominant IBS (IBSC). The overall IBS group scored higher on anxiety and depression than the control group. The IBSD and IBSC groups each had significantly higher scores for anxiety but did not significantly differ from the control group in scores for depression. There were no significant differences between the IBSD and IBSC groups in their cognitive appraisal of IBS symptoms. For the IBSD group, anxiety was significantly, positively correlated with commitment, effect, and threat, and depression was significantly, negatively correlated with controllability. In contrast, there were no significant correlations between mood and cognitive appraisal for the IBSC group. Multiple regression analyses with abdominal symptoms as dependent variables and cognitive appraisals as independent variables showed that for the IBSD group, abdominal pain was significantly, positively correlated with commitment, and abdominal discomfort was significantly, positively correlated with appraisal of effect and threat. For the IBSC group, abdominal pain and hard stool were significantly, positively correlated with commitment, and abdominal discomfort was significantly, positively correlated with appraisal of effect and threat.

**Conclusion:**

IBS patients as a general group report high levels of anxiety and depression. However, IBSD and IBSC were both associated only with high anxiety, but not depression, when compared to the non-IBS control group. For the IBSD group, anxiety was associated with cognitive appraisals, but this association was not found for the IBSC group. These groups did not differ in their associated cognitive appraisals, and are similar in terms of the positive relationship between abdominal pain and discomfort and the cognitive appraisals of coping.

## Introduction

Irritable bowel syndrome (IBS) is a common, costly, and potentially disabling gastrointestinal (GI) disorder characterized by abdominal pain/discomfort with altered bowel habits (e.g., diarrhea, constipation). The major pathophysiology of IBS are (1) abnormality of bowel movement, (2) reduction in bowel sensitivity thresholds, and (3) psychological abnormality [1]. Many IBS patients have psychological symptoms including depression, anxiety, tension, insomnia, frustration, hypochondria [2,3]. The onset and course of IBS are strongly influenced by psychosocial factors.

Researchers have investigated the relationship between cognitive factors and the treatment IBS. Toner et al [4] and have suggested that the cognitive-behavioral treatment approach has three major objectives: (1) to help clients reconceptualize their experience with IBS from helplessness and hopelessness to resourcefulness and optimism, (2) to help clients identify relationships among thoughts, feelings, behaviors, the environment, and IBS symptoms, (3) to empower clients to develop and implement increasingly more effective ways of coping with IBS in order to improve their quality of life. Research on the cognitive appraisal of IBS symptoms provides useful evidence for cognitive behavior therapy in more effective treatment for IBS. The biopsychological model of IBS of Drossman [1] includes the role of cognition in IBS symptoms. This model suggests that although IBS symptoms secondarily influence anxiety and depression, the physiological factors themselves influence the motor functions, sensory threshold, and stress reactivity of the gut. The cognitive appraisal of symptoms may be associated with negative emotions like anxiety or depression.

Lazarus and Folkman [5], who have contributed seminal research and conceptualizations for cognitive-behavioral therapy, emphasize that the cognitive appraisal of stressors has a strong effect on individual differences in stress responses. Cognitive appraisals involving "threat", "harmful effect", "challenge", "controllability", and others strongly regulate the selection of coping behavior and the extent of the stress response [6,7]. In regard to the relationship among cognitive appraisal, anxiety, and depression, some previous studies have suggested that challenge appraisal was associated with low depression and anxiety in multiple sclerosis patients [8] while threat appraisal was associated with anxiety and depression and challenge/controllability appraisal was associated with only anxiety [9]. Reduction of the appraisal of threat and improvement of controllability are generally thought to be important in stress management [10]. This may also be true for management of IBS. More detailed research is needed that investigates how individuals with IBS evaluate various IBS symptoms as stressors and what kinds of cognitive appraisals relate to anxiety and depression. Given not only the possibility that the degree of interference in daily life and disabling situations vary according to the type of abdominal symptom such as bowel movement but also the possibility of the difference of the relevant type of cognitive appraisal between anxiety and depression reported by Chandler's previous research [9], the type of cognitive appraisal of abdominal symptoms may vary according to the subtypes of IBS and the variation may be associated with the difference of anxiety among the subtypes of IBS.

Despite the need for psychological intervention, especially cognitive behavior therapy, there is little evidence of a relationship between cognitive appraisals like threat or controllability and negative moods like anxiety or depression in individuals with IBS. Furthermore, there have been no studies of the cognitive appraisal of IBS symptoms related to the subtypes of IBS. Research is needed not only using comparisons according to the presence or absence of IBS but also according to the subtype of IBS based on bowel movement disturbance [11]. Previous research reported that individuals with diarrhea-predominant IBS experienced anxiety more frequently than individuals with other types of IBS [12]. The pathological condition of IBS may differ according to the subtype of IBS both for physiological and psychological mechanisms, so it is indispensable to focus on the subtype of IBS.

We conducted a study of the relationship between the characteristic of cognitive appraisal of IBS symptoms and negative moods like anxiety or depression related to subtypes of IBS to contribute to the development of more effective psychological intervention.

### Hypothesis

1) There is difference in negative moods between the subtypes of IBS.

2) There is difference in cognitive appraisal of IBS symptoms between the subtypes of IBS.

3) There is difference in the pattern of correlation between cognitive appraisal of IBS symptoms and negative moods between the subtypes of IBS.

4) Although the severities of both diarrhea-predominant IBS and constipation-predominant IBS relate to cognitive appraisal of abdominal symptoms, the type of abdominal symptoms that affect cognitive appraisal or the type of cognitive appraisal of abdominal symptoms affected by abdominal symptoms vary according to the subtypes of IBS.

## Method

### Participants

A set of questionnaires was distributed to 1343 college students during classes.

### Measures

#### 1)Rome II Modular Questionnaire (RMQ; only items related to IBS) [3] translated into Japanese [13]

The Rome II diagnostic criteria for gastrointestinal disorder are widely used. IBS and its subtypes were defined according to the RMQ. The presence of IBS was indicated if participants had abdominal pain or discomfort during at least three weeks (at least once a week) in the last three months and 2 of 3 symptoms ((1) pain or discomfort getting better or stopping after a bowel movement, (2) a change in the number of bowel movements when the pain or discomfort starts, and (3) either softer or harder stools than usual when the pain or discomfort starts).

#### 2) Self-reported IBS Questionnaire (SIBSQ) [14,15]

SIBSQ was used to measure the severity of abdominal symptoms. It included 16 items rated from 1 to 7. Seven items of the SIBSQ related to diagnosis were used: abdominal pain, abdominal discomfort, change in bowel movement, loose stool, hard stool, urgency to move bowels, and constant urge to move bowels.

#### 3) Red-flag items

Seven red-flag items, based on the guidelines for IBS of the American Gastroenterological Association, were used to distinguish organic from functional gastrointestinal diseases. Individuals reporting one or more of these items were excluded from this study. These items were drastic weight loss, the participant's or a family history of organic bowel disease, history of digestive surgery, awakening by abdominal pain during night sleep, fever or arthralgia, blood in the stool, and anemia.

#### 4) Cognitive Appraisal Rating Scale (CARS) [16]

The CARS was used to measure how individuals with IBS usually rate themselves when they have IBS symptoms. The CARS is made up of four factors (commitment, appraisal of effect, appraisal of threat, and controllability) each assessed by two items rated on a scale from 1 to 4. "Commitment", "appraisal of effect", "appraisal of threat", and "controllability" of the subscales of CARS correspond to "challenge", "harmful effect", "threat", and "controllability" as the construct advocated by Lazarus & Folkman [5]. We asked the participants that only individuals who met the criteria for IBS respond to CARS.

#### 5) Hospital Anxiety and Depression Scale (HADS) [17,18]

The HADS is a self-report questionnaire consisting of 14 questions, comprising an anxiety subscale with seven items and a depression subscale with seven items. This psychometric instrument was chosen because all of its items solely refer to an emotional state and do not consider somatic symptoms.

### Procedure

We conducted the questionnaire survey with college students during classtime. We explained the research content and stipulated in writing and orally that the data would be analyzed statistically, the results would be published without individual identifiers, participation was voluntary and the students would not be penalized for non-cooperation We asked only those students who agreed to research cooperation after being informed of the above to complete the set of questionnaires. Moreover we conducted the research on an anonymous basis.

### Statistical Analysis

Data analysis was performed using SPSS 12.0 software. Comparison between groups on HADS score was done using analysis of variance (ANOVA). For cognitive appraisals and comparison between males and females, Student's t-tests were used. The relationship between cognitive appraisal and HADS was analyzed using Pearson's product-moment correlation coefficient. Multiple regression analysis (stepwise) was used to examine the effects of abdominal symptoms on cognitive appraisal of IBS symptoms. The significance level was set at less than 0.05.

## Results

### 1) Attributes of participants (see Table 1)

**Table 1:** Characteristics of participants

	Total	IBS	IBSD	IBSC	Control
N	1087	206	61	45	881
Male/Female	506/576	88/116	39/22	10/34	418/460
Age	19.72 ± 1.76	19.53 ± 1.87	19.90 ± 2.19	19.39 ± 1.43	19.77 ± 1.73

After eliminating individuals with red-flag items, invalid responses or incomplete questionnaires, valid data was obtained from 1087 participants (male: 506; female: 576; unidentified: 5). The mean age of the participants was 19.72 ± 1.76 (18 – 39) yrs. The participants included an 881 member control group who didn't meet the criteria for IBS (81.05%, male: 418; female: 460; unidentified: 5) and 206 individuals with IBS (18.95%; 88 males and 116 females). The participants consisted of 61 individuals with diarrhea-predominant IBS (IBSD, 39 males and 22 females), 45 with constipation-predominant IBS (IBSC, 10 males and 35 females), and 100 with IBS other than IBSD nor IBSC (39 males and 59 females). In the comparison of HADS, male participants had a significantly higher score than female participants on HADS-D (*t *[205] = 1.99, *p *< 0.05).

### 2) Comparisons of the mood (HADS) and cognitive appraisal scales (CARS; see Tables 2, 3)

**Table 2:** HADS scores

	IBSD	IBSC	Control		
					
	Mean Score	Mean Score	Mean Score	*F*	
HADS-A	7.56 ± 3.59**	7.27 ± 3.64*	5.85 ± 3.79	8.43	
HADS-D	5.66 ± 3.67	4.96 ± 2.95	4.63 ± 3.68	2.38	n.s.

** *p *> 0.01 (IBSD > Control)

* *p *> 0.05 (IBSC > Control)

n.s.= not significant

**Table 3:** CARS factors (cognitive appraisals of IBS symptoms)

	IBSD	IBSC		
	Mean Score	Mean Score	*t*	
Commitment	4.22 ± 1.58	4.53 ± 1.69	0.90	n.s.
Appraisal of Effect	3.88 ± 1.72	4.20 ± 1.62	0.90	n.s.
Appraisal of Threat	3.39 ± 1.91	3.20 ± 1.42	0.53	n.s.
Controllability	4.63 ± 1.57	4.40 ± 1.75	0.65	n.s.

n.s.= not significant

Scores on the HADS-A and D were compared related to the presence of IBS. Individuals with IBS had significant higher scores on the HADS (HADS-A: *t *[1086] = 5.26, *p *< 0.001; HADS-D: *t *[1086] = 2.48, *p *< 0.05) than the control group. In the comparison among the control group, IBSD and IBSC, there was a significant main effect for the HADS-A (*F *[2,285] = 8.43, *p *< 0.001), but not for HADS-D. The IBSD group and the IBSC group each had significant higher scores for anxiety symptoms than the control group.

There were no significant differences between the IBSD and IBSC groups in the Cognitive Appraisal Rating Scale's subscales (CARS subscales: commitment, appraisal of effect, appraisal of threat, and controllability).

### 3) Relationship between cognitive appraisal of IBS symptoms (CARS) and anxiety and depression (HADS; see Table 4)

**Table 4:** Correlations between CARS factors and HADS scores

	IBSD		IBSC	
		
	HADS-A	HADS-D	HADS-A	HADS-D
Commitment	0.31 *	0.05	0.21	0.10
Appraisal of Effect	0.32 *	0.06	0.27	0.24
Appraisal of Threat	0.31 *	-0.03	0.19	0.13
Controllability	-0.22	-0.34 *	0.00	-0.17

* *p *> 0.05

For the IBSD group, the HADS-A significantly correlated with commitment (*r *= 0.31, *p *< 0.05), appraisal of effect (*r *= 0.32, *p *< 0.05), and appraisal of threat (*r *= 0.31, *p *< 0.05), and the HADS-D significantly correlated with controllability (*r*=-0.34, *p *< 0.05). In contrast, there were no significant correlations for the IBSC group.

### 4) Relationship of abdominal symptoms (SIBSQ) to cognitive appraisal of IBS symptoms (CARS; see Table 5)

**Table 5:** Correlations of SIBSQ symptoms with CARS factors

**IBSD**	Commitment		Appraisal of Effect		Appraisal of Threat		Controllability	
	*β*	*r*	*β*	*r*	*β*	*r*	*β*	*r*

Abdominal Pain	0.59 ***	0.59 ***	-	0.44 ***	-	0.37 **	-	-0.03
Abdominal Discomfort	-	0.57 ***	0.47 ***	0.47 ***	0.40 **	0.40 **	-	-0.11
Change in Bowel Movement	-	0.48 **	-	0.38 **	-	0.37 **	-	0.05
Loose Stool	-	0.30 *	-	0.23	-	0.17	-	0.07
Urgency to Move Bowels	-	0.18	-	0.17	-	0.05	-	0.03

	*R*^2 ^= 0.35 ***		*R*^2 ^= 0.22 ***		*R*^2 ^= 0.16 **			

**IBSC**	Commitment		Appraisal of Effect		Appraisal of Threat		Controllability	

	*β*	*r*	*β*	*r*	*β*	*r*	*β*	*r*

Abdominal Pain	0.47 **	0.32 *	-	0.21	-	0.38 **	-	-0.10
Abdominal Discomfort	-	0.62 ***	0.52 ***	0.52 ***	0.39**	0.39 **	-	-0.07
Change in Bowel Movement	-	0.38 **	-	0.13	-	0.18	-	-0.09
Hard Stool	0.33 *	0.54 ***	-	0.36 *	-	0.32 *	-	-0.06
Constant Urge to Move Bowels	-	0.39 **	-	0.44 **	-	0.31 *	-	-0.02

	*R*^2 ^= 0.47 ***		*R*^2 ^= 0.27 ***		*R*^2 ^= 0.15 *			

β = standardized partial regression coefficient

*r *= correlation coefficient

*R*^2 ^= multiple correlation coefficient

* *p *< 0.05 ** *p *< 0.01 *** *p *< 0.001

We performed a multiple regression analysis to examine the effect of abdominal symptoms on cognitive appraisal of IBS symptoms, with the abdominal symptoms assessed by the SIBSQ as dependent variables and the subscale scores of the CARS as independent variables. For the IBSD group, abdominal pain significantly correlated with commitment (*β *= 0.59, *p *< 0.001), and abdominal discomfort significantly correlated with appraisals of effect (*β *= 0.47, *p *< 0.001) and threat (*β *= 0.40, *p *< 0.01). For the IBSC group, abdominal pain (*β *= 0.47, *p *< 0.01) and hard stool (*β *= 0.33, *p *< 0.05) significantly correlated with commitment, and abdominal discomfort significantly correlated with appraisals of effect (*β *= 0.52, *p *< 0.001) and threat (*β *= 0.39, *p *< 0.01).

## Discussion

The attributes of the participants in the present study are similar to those of previous studies. There were more males than females with IBSD, while the sex ratio was reversed for IBSC.

The results of the present study suggest that individuals with IBSD show a more prominent relationship between cognitive appraisal of abdominal symptoms and negative mood than individuals with IBSC. Both the IBSD and IBSC groups had higher anxiety than the control group, although there were no significant differences for depression. There were no significant differences between the groups with IBSD and IBSC in the scores for each of the cognitive appraisal factors. However, in the IBSD group only, anxiety was correlated with commitment, effect and threat, and depression was correlated with controllability. In contrast, anxiety in the IBSC group did not relate to their cognitive appraisals, so there is a possibility that depression in the IBSC group affects constipation symptoms. These results suggest that for the IBSD group, an improvement in anxiety may occur through intervention addressing cognitive appraisals of abdominal symptoms. Although there was no significant difference between IBSD and IBSC in HADS or CARS score, the IBSD group showed a more prominent difference than the IBSC group in comparison with the control group. Therefore, our first and second hypotheses were not confirmed in the main. As previous research [19] suggested that the subtypes of IBS alter with time, there is a possibility that the same was true in the participants in this study. In contrast, as the results of this study suggested that there is difference between the subtypes of IBS in the pattern of correlation between cognitive appraisal of IBS symptoms and negative moods, our third hypothesis was confirmed.

In the present study, not only estimations of increasing threat and the effect or low controllability of abdominal symptoms in IBSD but also the attitude of improving their abdominal symptoms was related to negative emotions. This result shows the possibility that when abdominal symptoms are not improved despite the intention to improve them, then anxiety is increased. Our previous research on panic disorder patients with IBS suggested that panic disorder patients with diarrhea-predominant IBS showed a tendency toward a higher frequency of IBS preceding panic disorder episodes than panic disorder patients with constipation-predominant IBS [20]. It is possible that high anxiety in individuals with constipation-predominant IBS does not get worse related to cognitive appraisal for abdominal symptoms, but instead the high anxiety may cause their constipation. The results of the present study may partially correspond with this result of our previous study.

For both the IBSD and IBSC groups, the multiple regression analysis showed that the severity of abdominal pain or discomfort was related to the degree of commitment, appraisal of effect, and appraisal of threat. Moreover hard stool in IBSC was related to commitment for IBS symptoms. Both the IBSD and IBSC groups showed that the abdominal symptoms related to a change in bowel movement was not related to the cognitive appraisal of abdominal symptoms. Controllability was independent of the severity of abdominal symptoms. Controllability may relate to psychological factors that are not specific to IBS. Although there were minor differences in the type of abdominal symptoms that affect cognitive appraisal according to the subtypes of IBS, our fourth hypothesis was partially confirmed.

Although this study provided some new insight, there were three limitations. First, the severity of abdominal symptoms in the general population of our sample may differ from clinical patients. Although there was no significant difference in depression, individuals with IBSD showed higher depression scores than IBSC individuals; the p-value was 0.10. It is possible that the finding of no significant difference for depression was due to the fact that the participants in the present study were not a clinical sample. Second, the participants in this study were college students. Therefore the results of this study do not necessarily apply to individuals with IBS of all ages. In the future, we should investigate the psychological characteristics of IBS among people of all ages. Third, many psychological differences in IBS between males and females were found in previous studies [21]. In the present research, males had a higher HADS-D score than females. Although there is significant difference between IBSD and IBSC in HADS-D score, there is a possibility that the difference in gender and the difference of sex ratio between IBSD and IBSC had an effect on the results of this study. We should do further exploration in larger samples, and even out the discrepancy between the number of male and female participants.

## Conclusion

Persons with IBS as a general group report high levels of anxiety and depression. However, IBSD and IBSC were both associated only with high anxiety, but not depression, compared to the non-IBS control group. For the IBSD group, anxiety was associated with cognitive appraisals, but this association was not found for the IBSC group. Therefore, it was suggested that although IBSD and IBSC share a relationship between the severity of abdominal symptoms and cognitive appraisal for IBS symptoms, they differ in the relationship between negative emotions and cognitive appraisal for IBS symptoms. These groups did not differ in their associated cognitive appraisals, and are similar in terms of the positive relationship between abdominal pain and discomfort and the cognitive appraisals of coping. Considering the subtype of IBS is indispensable for managing IBS-related cognitive factors in clinical practice or research.
